# Exosomal Lipids Impact Notch Signaling and Induce Death of Human Pancreatic Tumoral SOJ-6 Cells

**DOI:** 10.1371/journal.pone.0047480

**Published:** 2012-10-19

**Authors:** Sadia Beloribi, Elodie Ristorcelli, Gilles Breuzard, Françoise Silvy, Justine Bertrand-Michel, Evelyne Beraud, Alain Verine, Dominique Lombardo

**Affiliations:** 1 Center for Research in Oncobiology and Oncopharmacology (CRO2), Aix-Marseille Université, Marseille, France; 2 UMR 911, INSERM, Marseille, France; 3 Lipidomic Core Facility, INSERM-IFR-BMT-MetaToul, CHU Purpan, Toulouse, France; Deutsches Krebsforschungszentrum, Germany

## Abstract

Exosomes are of increasing interest as alternative mode of cell-to-cell communication. We previously reported that exosomes secreted by human SOJ-6 pancreatic tumor cells induce (glyco)protein ligand-independent cell death and inhibit Notch-1 pathway, this latter being particularly active during carcinogenesis and in cancer stem cells. Therefore, we asked whether exosomal lipids were key-elements for cell death and hypothesized that cholesterol-rich membrane microdomains were privileged sites of exosome interactions with tumor cells. To address these questions and based on the lipid composition of exosomes from SOJ-6 cells (Ristorcelli *et al*. (2008) FASEB J. 22; 3358–3369) enriched in cholesterol and sphingomyelin (lipids forming liquid-ordered phase, Lo) and depleted in phospholipids (lipids forming liquid-disordered phase, Ld), we designed Synthetic Exosome-Like Nanoparticles (SELN) with ratios Lo/Ld from 3.0 to 6.0 framing that of SOJ-6 cell exosomes. SELN decreased tumor cell survival, the higher the Lo/Ld ratio, the lower the cell survival. This decreased survival was due to activation of cell death with inhibition of Notch pathway. FRET analyses indicated fusions/exchanges of SELN with cell membranes. Fluorescent SELN co-localized with the ganglioside GM1 then with Rab5A, markers of lipid microdomains and of early endosomes, respectively. These interactions occurred at lipid microdomains of plasma and/or endosome membranes where the Notch-1 pathway matures. We thus demonstrated a major role for lipids in interactions between SELN and tumor cells, and in the ensued cell death. To our knowledge this is the first report on such effects of lipidic nanoparticles on tumor cell behavior. This may have implications in tumor progression.

## Introduction

Exosomes are small vesicles released by many cell types. Since their first description in reticulocytes as carbage bags, there is compelling evidence for exosome secretion by many hematopoietic and non-hematopoietic cell types [1]. Exosomes are formed by invagination of the limiting membrane of late endosomes, which results in the formation of multivesicular bodies. Exosomes are released in the extracellular space upon fusion of multivesicular bodies with the plasma membrane. The biological significance of exosomes is largely questioned [2]. Because of the abundance of signaling proteins and adhesion molecules at the surface of exosomes [3], it is hypothesized that exosomes may serve as vehicles for long-range intercellular communication. This notion is supported by the presence of these vesicular structures in human blood [4] where they act as “multi-purpose carriers” in cell communication [5]. Furthermore they might have complex roles in disease with regard to communication [6], protection [7] and exchange of genetic information [8]. Upon interaction with target cell, exosomes may deliver their information in lipid-forming microdomains (termed lipid-raft) dependent mechanism [9]. The transfer of proteins or of genetic material and the subsequent biological effects on cell fate are well described [1], [3], [4]. However impacts of exosomal lipids, which are also transferred [10], on cell behavior remain largely ignored.

We recently reported that the human pancreatic cancer SOJ-6 cells expressed exosomal nanoparticles, referred to as exosomes, rich in lipid-forming rafts [11]. These exosomes decreased tumor cell proliferation through mitochondria-dependent cell death. Cell susceptibility to death induced by exosomes seems to be inversely correlated with the expression levels of partners of the Notch-1 survival pathway such as ADAM17 and ICN [12]. Moreover exosomes interacted with SOJ-6 cells to disturb the functioning of partners in the Notch-1 survival pathway localized in membrane lipid microdomains, in particular the γ-secretase complex which is sensitive to its lipid microenvironment [13]. These effects on Notch functioning down-regulate the phosphorylation of the pro-apoptotic Phosphatase and tensin homolog deleted on chromosome 10 (PTEN) and Glycogen Synthase Kinase 3β (GSK-3β) leading to their activation. Lastly, GSK-3β inhibits mitochondrial pyruvate dehydrogenase, up-regulates pro-apoptotic Bax protein, down-regulates anti-apoptotic Bcl-2, Hes-1(a nuclear target of the Notch pathway) and cyclin D1 expressions, and promotes arrest of the cell cycle in the G_0_–G_1_ transition phase. We therefore demonstrated that the interaction of exosomes with tumor cells conspicuously involves Notch signaling to drive target cells towards apoptosis *via* the intrinsic pathway [12].

The present study aims at identifying the key-elements involved in the death of pancreatic tumor cells upon exosomes challenging. Because exosome lipids seem to be required for their capture [11], [14], we get attention to lipid components of exosomes and based on the lipid composition of cell death-inducing exosomes expressed by SOJ-6 cells in which lipids forming raft domains dominated, we yielded Synthetic Exosome-Like Nanoparticles (SELN) with similar lipid composition but lacking proteins, which are not involved in the observed effects of exosomes on cell proliferation [12]. We here demonstrated that SELN were able to trigger inhibition of tumor SOJ-6 cells proliferation through the mitochondria-dependent cell apoptotic pathway, as already described with exosomes [12]. In SOJ-6 cells, SELN rich in lipid-forming raft microdomains down-regulated the phosphorylation of pro-apoptotic PTEN and GSK-3β, leading to their activation. These SELN also decreased the expression of anti-apoptotic Bcl-2, meanwhile increasing that of pro-apoptotic Bax proteins. Furthermore SELN rich in lipid-forming microdomains decreased the amount of intracellular domain of Notch (ICN), which consecutively decreased the expression of Hes-1, the nuclear target of ICN. Lipid-dependent apoptotic pathways such as the ceramide death pathway or the endoplasmic stress due to cholesterol loading are likely not involved. Such activation of apoptotic pathway is not implicated in SELN-insensitive human pancreatic tumor MiaPaCa-2 cells. We also showed that SELN interacted with lipid microdomains of cell membranes where they co-localize with the ganglioside GM1, Rab5A and Notch-1. Therefore, SELN impact on lipid microdomains where Notch-1 signaling concentrates thus confering a unique role to lipids of exosomes.

## Results

### Synthesis of Exosome-like Nanoparticles

**Figure 1:**
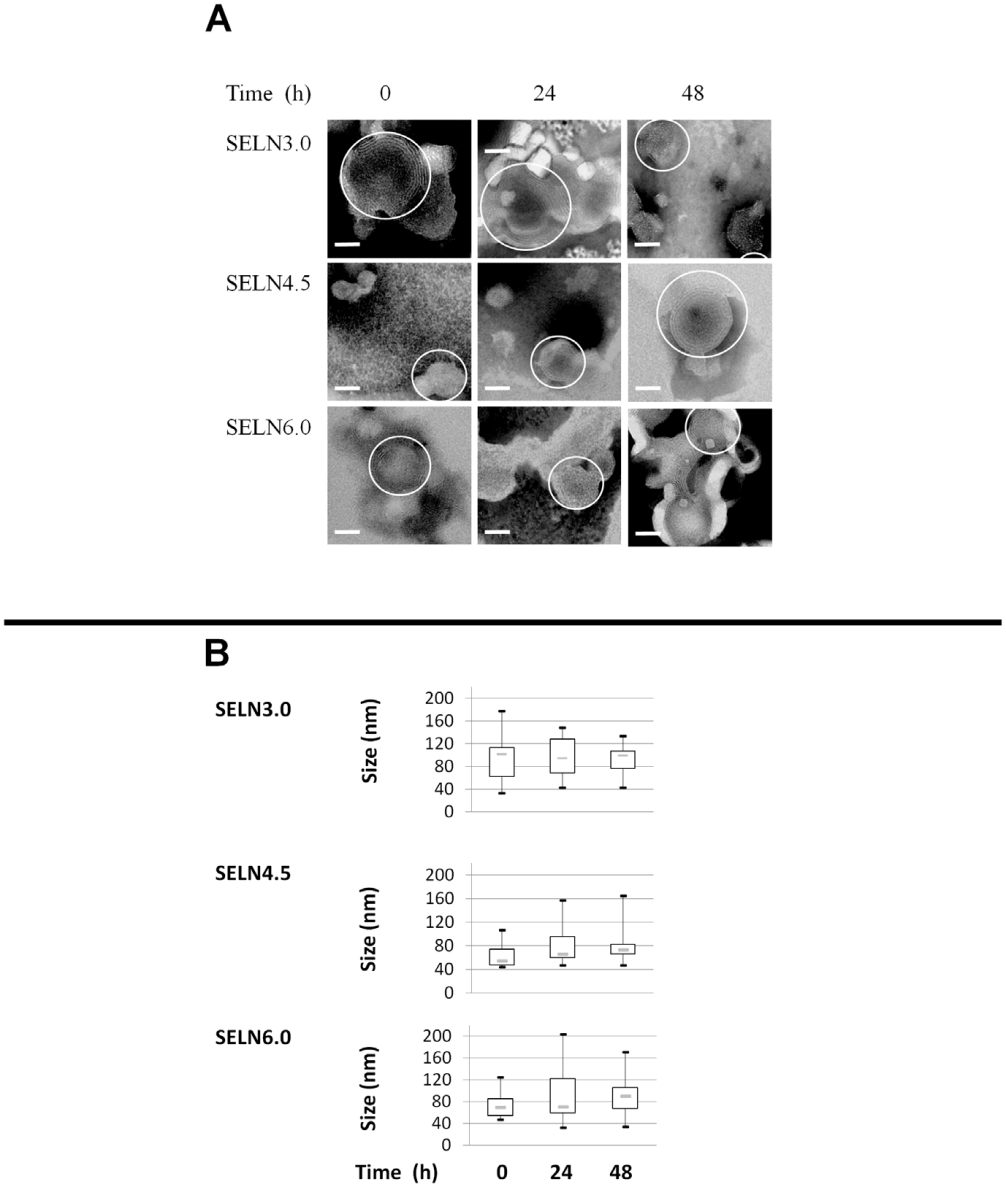
Synthetic exosome-like nanoparticles (SELN). (A) Synthetic exosome-like nanoparticles (SELN) were examined by electron microscopy. SELN3.0, SELN4.5 and SELN6.0 preparations were incubated at room temperature, then 3 µl were removed at 0, 24 h and 48 h. SELN were then disposed on top of Formvar-coated 300-mesh carbon grids and treated with 2% phosphotungstic acid. Several fields were photographed and used to determine the diameter of SELN. Scale bars on microphotographs represent 50 nm. (B) The range of observed diameters for lipid structures as represented in A was statistically represented by the box plot. The dashed line inside the box represents the median diameter of SELN (n = 20). The box represents the interquartile range (50% of values). Tails extend to values within 1.5 times the interquartile range.

We hypothesized that lipids were key-elements in cell death induced by exosomes expressed by pancreatic tumor cells [11], [12]. Examination of the lipid composition of cell death-promoting exosomes isolated from SOJ-6 pancreatic cancer cells ([11], see **Table 1** **)**, showed that lipids forming liquid ordered phase (Lo) and involved in membrane lipid microdomains termed rafts, predominate as they represent some 79% of total exosome lipids. The remaining lipids were phospholipids forming liquid disordered phase (Ld). Consequently, the ratio Lo over Ld (Lo/Ld) in exosomes originating from SOJ-6 cells was about 4.1 [11]. Therefore to confirm our hypothesis concerning the role of lipids, in part that of lipid microdomains, in cell death promoted by exosomes [11], [12], we mimicked the lipid composition of exosomes from SOJ-6 cells and mixed commercial lipid solutions to prepare synthetic lipid particles with increasing theoretical ratios Lo/Ld of 3.0, 4.5 and 6.0 (see Materials and Methods). These ratios were selected to allow us to frame the ratio Lo/Ld of cell death-promoting exosomes from SOJ-6 cells (see **Table 1**) and to determine whether lipid-forming microdomains are actually key elements in cell death promoted by pancreatic cancer cell exosomes. At the end of the synthesis we examined the ratio cholesterol over phosphatidylcholine (cholesterol and PC are highly represented in Lo and Ld phases, respectively) of these synthetic particles to check that required ratios after commercial lipid mixing were practically obtained. **Table 1** showed that molar ratios cholesterol over phosphatidylcholine were quite similar in starting lipid mixtures (cholesterol/PC, theoretical, **Table 1**) than in isolated synthetic particles (cholesterol/PC, practical) determined by lipid analyses. These Synthetic Exosome-Like Nanoparticles or SELN will be referred to as SELN3.0, SELN4.5 and SELN6.0, respectively. Based on SELN labeling (see below) the yield of preparation was 56.5±2.9%. The density of SELN determined by sucrose density gradient ranges from 1.065 to 1.085 (data not shown). These structures can be monolamellar or multilamellar (onion peels, **Fig. 1A**). The median diameter determined by electron microscopy ranges between 55 nm to 100 nm and did not change with time (**Fig. 1B**), which agrees with the stability with time of natural exosomes [15]. Density and size of SELN correlate with those of cell-expressed exosomes [4].

**Table 1:** Lipid composition of synthetic exosome-like nanoparticles (SELN).

	Exosomes (SOJ-6)*		SELN						
			SELN3.0			SELN4.5		SELN6.0	
	% Total lipid (weight)	nmol		% Total lipid (weight)	nmol	% Total lipid (weight)	nmol	% Total lipid (weight)	nmol
Sphingomyeline	14.20	40.35		14.03	40.35	15.31	40.35	16.04	40.35
Ceramide	0.50	1.26		0.44	1.26	0.48	1.26	0.50	1.26
Cholesterol	59.80	159.56		55.31	159.00	60.33	159.00	63.98	159.00
Cholesteryl oleate	4.50	10.62		3.68	10.60	4.02	10.60	4.21	10.60
Phosphatidylcholine (PC)	12.10	38.10		17.22	49.50	12.52	33.00	9.82	24.70
Phosphatidylethanolamine	2.40	4.85		2.19	6.30	1.59	4.20	1.25	3.15
Phosphatidylserine	1.90	6.43		2.92	8.40	2.11	5.57	1.67	4.20
Lysophosphatidylcholine	2.80	5.70		2.28	7.41	1.87	4.93	1.47	3.70
Trioleyl glycerol	2.00	4.63		1.61	4.63	1.76	4.63	1.84	4.63
								
Lipid forming Lo phase	79.00	211.79		75.09	215.84	81.90	215.84	85.79	215.84
Lipid forming Ld phase	19.20	55.08		24.91	71.61	18.10	47.69	14.21	35.75
**Raft lipids over non raft lipids**	**4.11**			**3.01**		**4.53**		**6.04**	
Cholesterol/PC theoretical**					3.21		4.82		6.51
Cholesterol/PC practical***		4.19			3.50		4.85		5.83

* Data from ref. 11, given for comparison.

** Ratio in starting lipid mix to prepare SELN of various raft lipids over non-raft lipids ratios.

*** Ratio in SELN after isolation, data are average of two independent assays.

### Effects of SELN on Cell Survival

**Figure 2:**
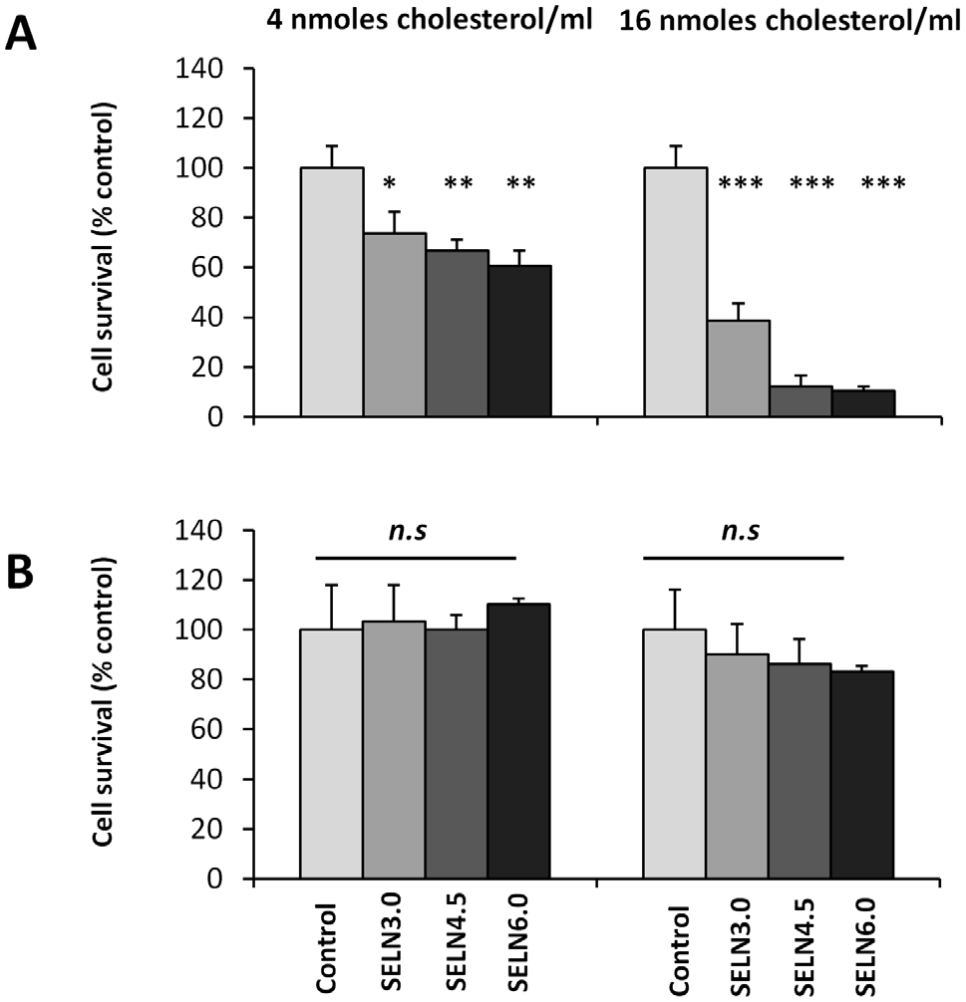
Survival of SOJ-6 and MiaPaCa-2 cells in the presence of SELN. SOJ-6 (A) and MiaPaCa-2 (B) cells were starved and incubated for 24 h with SELN (left panel; 4 nmoles of cholesterol/ml, right panel; 16 nmoles of cholesterol/ml). Cell survival was determined by MTT assay. Results are mean (± SD) of independent experiments (n = 32, Student’s *t*-test).

To determine whether lipids may be key-elements of exosomes in promoting the inhibition of cell survival, MiaPaCa-2 and SOJ-6 cells were challenged with SELN (**Fig. 2**). Two doses of SELN, corresponding to 4 nmoles cholesterol/ml and 16 nmoles cholesterol/ml were tested. The higher amount of cholesterol (determined using SELN labeled with [^3^H]-cholesterol) corresponds to that found in exosomes from SOJ-6 cells when used at 5 µg/ml in term of proteins allowing significant cell proliferation inhibition [12]. As expected the higher dose of SELN is more effective in SOJ-6 cell survival inhibition than the lower dose (**Fig. 2A**). Data indicate that higher was the ratio Lo/Ld, higher was the inhibition in SOJ-6 cell proliferation. However, the proliferation of MiaPaCa-2 cells was unaffected independently of the ratio and the dose applied (**Fig. 2B**). These data clearly demonstrate the lack of toxic effects of cholesterol at 16 µM. Giving the similar effects of SELN4.5 and SELN6.0, we focused on the latter particles and compared their effects to those of SELN3.0, this allows us to make a clear cut between SELN rich in lipid forming microdomains (SELN6.0) and SELN poor in these lipid structures (SELN3.0).

### Effects of SELN on the Notch-1 Survival Pathway

**Figure 3:**
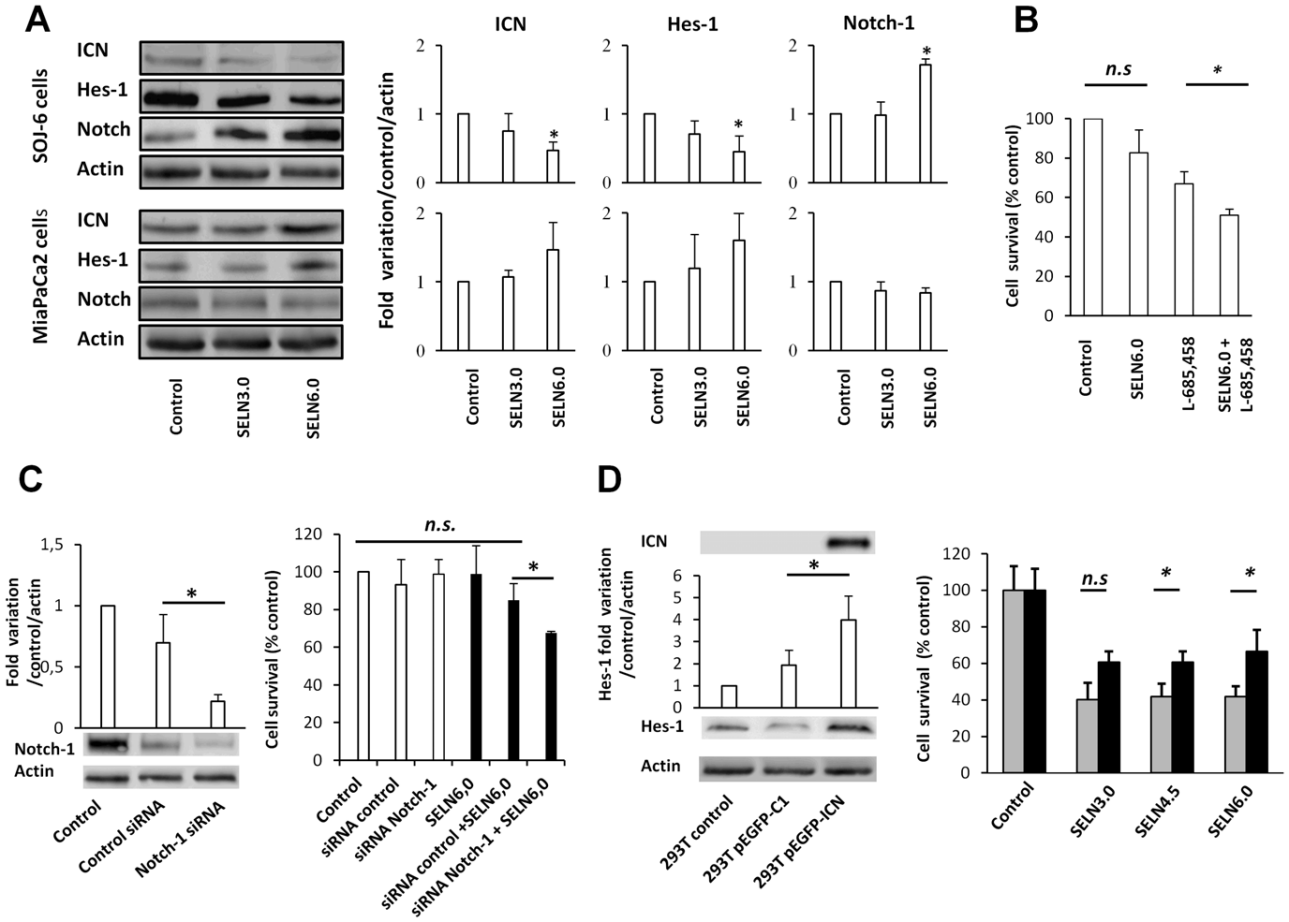
Effect of SELN on Notch pathway. (A) SOJ-6 and MiaPaCa-2 cells were starved then treated for 24 h with SELN (16 nmoles cholesterol/ml) and lysed. Cell lysate proteins were separated on SDS-PAGE (80 µg of proteins/lane) and electrotransferred onto nitrocellulose membrane. The levels of ICN, Hes-1, Notch-1 and β-actin were determined by probing membranes with specific antibodies as indicated. Western blots are representative of three independent experiments. Lane 1, control performed in the absence of SELN, Lane 2, in the presence of SELN 3.0, Lane 3, in the presence of SELN 6.0. The right panels in figure 3A represents the quantification of western blots (means (± SD) of three independent experiments) using the NIH Image program (Mann-Whitney test). (B) MiaPaCa-2 cells were incubated with medium (control), with L-685,458 γ-secretase inhibitor (GSI, 2.5 µM), with SELN6.0 (16 nmoles of cholesterol/ml), with SELN6.0 and GSI. The L-685,458 GSI was added 60 min before freshly prepared SELN6.0. Cells were further incubated for 24 h in the presence of each component and cell proliferation was determined. Results are means (± SD) of independent experiments (n = 36, Student’s *t*-test). (C) MiaPaCa2 cells were transfected with a mix of Notch-1 siRNA or with a control siRNA. In the left panel cell lysate proteins (50 µg of proteins) of parental, control and Notch-1 siRNA transfected MiaPaCa-2 cells were separated on SDS-PAGE and analyzed by western blotting (72 h post-transfection). The histogram indicates quantification of western blots. In the right panel cell survival of parental and transfected MiaPaCa-2 cells was assessed through a MTT test after 24 hours of incubation with (black columns) or without (white columns) SELN6.0. The proliferation of MiaPaCa-2 cells recorded in the absence of SELN was taken as 100%. Results are means (± SD) of three independent experiments (Mann-Whitney test). (D, left panel) HEK 293 T cells were transiently transfected with the pEGFP-C1 control plasmid or pEGFP-ICN plasmid encoding ICN (Notch-1 intracellular domain). Cell lysate proteins of parental and transfected HEK 293T cells were separated on SDS-PAGE and analyzed by western blotting using primary antibodies as indicated. Western blotting replicates depicted the expression level of ICN and the nuclear target of ICN, Hes-1, in parental (293T control), pEGFP-C1 control vector-transfected (293TpEGFP-C1) and pEGFP-ICN-transfected (293TpEGFP-ICN) HEK 293T cells. The amount of loaded proteins was the same (50 µg) for each lane as indicated by β-actin probing. The histogram displays the quantification of Hes-1 as determined from western blots. (D, right panel) HEK 293TpEGFP-C1-transfected cells (grey columns) and HEK 293TpEGFP-ICN-transfected cells (black columns) were challenged without (control) or with SELN3.0, SELN4.5 and SELN6.0 (16 nmoles cholesterol/ml) for 24 h. Cell survival was determined with MTT. The proliferation of HEK 293T cells transfected with the control vector (293TpEGFP-C1) recorded in the absence of SELN was taken as 100%. Values are means ± SD of three independent experiments (Mann-Whitney test).

To confirm that SELN as exosomes [12] impact on the Notch pathway signaling to induce cell death, SOJ-6 cells were challenged with SELN3.0, and 6.0 (16 nmoles cholesterol/ml) for 24 h and lysed to examine protein expression profile. As shown on **Fig. 3A**, ICN (Notch-1 intracellular domain) expression was significantly decreased in lysates upon SOJ-6 cells treatment with SELN6.0. Consequently Hes-1 expression was also significantly decreased meanwhile that of Notch-1 was increased. Note that SELN3.0 had no significant effect on the expression of ICN, Hes-1 and Notch-1 in SOJ-6 cells. Furthermore, the expression of Notch-1, ICN and Hes-1 was also not significantly affected in MiaPaCa-2 cells challenged with SELN whatever their composition (**Fig. 3A**). These data suggested that the Notch survival pathway of SOJ-6 cells was affected by SELN, in part SELN with a high ratio Lo/Ld. We have hypothesized that MiaPaCa-2 cells were resistant to exosomes because of the high expression level of Notch pathway partners [12]. To confirm this hypothesis we have modulated the Notch pathway efficiency in SELN resistant MiaPaCa-2 cells. For this purpose we used the γ-secretase inhibitor L-685,458 (GSI) at concentration (2.5×10^−6^ M) below its IC_50_ on MiaPaCa-2 cells (IC_50_ = 14 (±4)×10^−6^ M, see **Fig. S1**) to only allow a decrease in Notch pathway efficiency. As shown in **Fig. 3B** the γ-secretase inhibitor makes MiaPaCa-2 cells significantly sensitive to SELN6.0. We further invalidated the expression of Notch-1 in MiaPaCa-2 cells using a specific mix of siRNA. As shown in **Fig. 3C** (left panel), following siRNA (100 nM) transfection the extinction of Notch-1 expression was effective at least for 72 h. However and albeit partial, the invalidation of the expression of the Notch-1 survival pathway significantly affects the sensitivity of MiaPaCa-2 cells to SELN6.0 (right panel).

Finally and to reverse the inhibitor effects of SELN6.0 on Notch signaling, we attempted to (over-)express ICN, the dominant active intracellular form of Notch-1 receptor independent of ligand binding in exosome-sensitive pancreatic SOJ-6 cells expressing low level of Notch partners [12]. Because we obtained low transfection level of SOJ-6 cells with the plasmid encoding ICN, transient transfection was yielded in the HEK 293T cell line. Western blot (**Fig. 3D**) indicates that HEK 293T cells transfected with pEGFP-ICN (293T-pEGFP-ICN) expressed ICN, when compared to parental HEK 293T cells (293T control) and to pEGFP-C1 control vector-transfected cells (293T-pEGFP-C1). Expression of ICN lead to the over-expression of Hes-1 (**Fig. 3D**, left panel **)** meaning that ICN reaches the nucleus to induce the expression of target genes**.** HEK 293T cells transfected with the pEGFP-C1 control vector were sensitive to SELN (**Fig. 3D**, right panel). ICN expression and consecutive Hes-1 over-expression did not affect cell survival of the controls. In spite of conditions in which transfection efficiency remained rather low (60%), ICN and Hes-1 (over)-expression allowed significant reversion of SELN effects on cell survival (**Fig. 3D**, right panel **)**. Note that SELN3.0 were less efficient than SELN4.5 and SELN6.0 in reverting the effect of ICN and Hes-1 (over)-expression. Consequently, SELN, like exosomes expressed by pancreatic cancer cells, in part exosomes expressed by SOJ-6 cells [11], [12] affected the functioning of the Notch-1 survival pathway.

### Effects of SELN on SOJ-6 Cell Death

**Figure 4:**
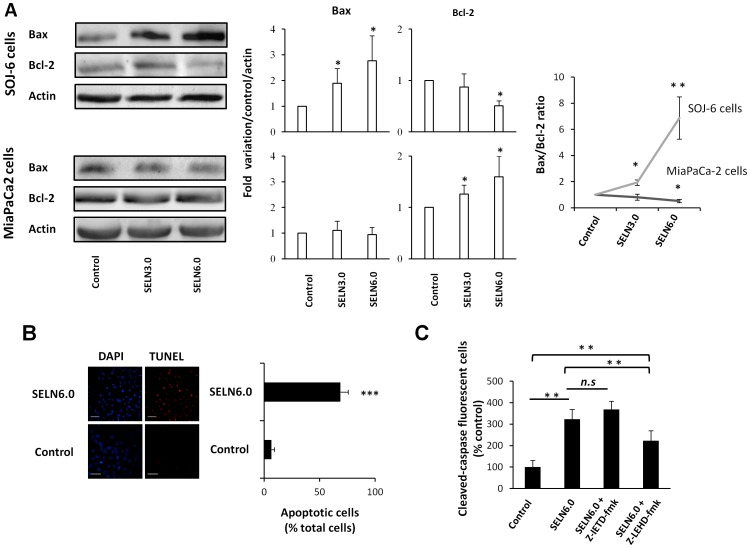
Effect of SELN on apoptosis. (A) SOJ-6 and MiaPaCa-2 cells were starved then treated for 24h with SELN (16 nmoles cholesterol/ml) and lysed. Cell lysate proteins were separated on SDS-PAGE (80 µg of proteins/lane) and electrotransferred onto nitrocellulose membrane. The levels of Bax, Bcl-2 and β-actin were determined by probing membranes with specific antibodies as indicated. Western blots are representative of three independent experiments. Lane 1; control performed in the absence of SELN, Lane 2; in the presence of SELN 3.0, Lane 3; in the presence of SELN 6.0. The right panels in figure 4A represents the quantification of western blots and the ratio Bax/Bcl-2. Data are means (± SD) of three independent experiments using the NIH Image program (Mann-Whitney test). (B) Apoptosis was determined using the Terminal Transferase dUTP Nick End Labeling (TUNEL). SOJ-6 cells were starved then treated with SELN6.0 (16 nmoles cholesterol/ml) for 24h then fragmented DNA was stained according to the protocol given with the ApopTag® Red In Situ Apoptosis Detection Kit (right pictures). Nuclei were counterstained with DAPI (left pictures). Apoptotic cells were visualized under a Zeiss fluorescence microscope equipped with a digital camera. The ratio of apoptotic cells to total cells was counted in 5-to-10 random area, in both control and SELN6.0 treated SOJ-6 cells (right histogram, (means (± SD) of three independent experiments, Mann-Whitney test), scale bar = 100 µm. (C) SOJ-6 cells were incubated 4 h with medium (control), with SELN6.0 (16 nmoles of cholesterol/ml, column 2), with SELN6.0 and Z-IETD-fmk (a caspase-8 inhibitor, 10 µM, column 3) or Z-LEHD-fmk (a caspase-9 inhibitor, 10 µM, column 4). After adding freshly prepared SELN6.0, cells were incubated for another 24h. After washing and fixation, cleaved caspase-positive cells were counted under fluorescent microscopy. Results are means (± SD) of fluorescent cell amounts collected in 10 fields per assay (at least 3 independent assays). Results are expressed as percentage of cleaved caspase-positive cells relatively to controls (Mann-Whitney test).

We have previously shown that cell treatment by natural exosomes increased the pro-apoptotic Bax protein expression detrimental to that of Bcl-2 anti-apoptotic protein, meaning that the intrinsic apoptotic pathway may be activated by exosomes [11], [12]. Therefore lysates of cells treated with SELN were also examined for Bax and Bcl-2 expression. **Fig. 4A** indicates that Bax expression was significantly increased in SOJ-6 cells challenged with SELN6.0 detrimental to that of Bcl-2 protein. These two proteins were also affected to a lower extent in cells challenged with SELN3.0. Bax was not affected in MiaPaCa-2 cells upon challenging with SELN6.0 or SELN3.0. Surprisingly the anti-apoptotic Bcl-2 protein was over-expressed in MiaPaCa-2 cells upon SELN challenging. Therefore the balance between pro-apoptotic Bax over anti-apoptotic Bcl-2 proteins (Bax/Bcl-2 ratio) strongly favored apoptosis activation in SOJ-6 cells upon SELN6.0 challenging (**Fig. 4A** **)**. Consistent with this, TUNEL evaluation of DNA fragmentation significantly increased in SOJ-6 cells treated with SELN6.0 (**Fig. 4B** **)** and correlated with the decrease in SOJ-6 cell proliferation (see **Fig**. **2A**). To further determine which cell apoptotic pathway is activated upon SOJ-6 cells treatment with SELN6.0, a study was performed using caspase inhibitors. The amount of cleaved caspase fluorescent cells was significantly increased in the presence of SELN6.0 compared to mock-treated cells (**Fig. 4C**). When compared to SELN6.0-treated cells, the inhibitor of caspase-8 (Z-IETD-fmk) did not affect the amount of cleaved caspase fluorescent cells, in contrast to the inhibitor of caspase-9 (Z-LEHD-fmk) (**Fig. 4C**). Taken as a whole these data strongly suggested that the intrinsic apoptotic pathway is activated by SELN6.0.

**Figure 5:**
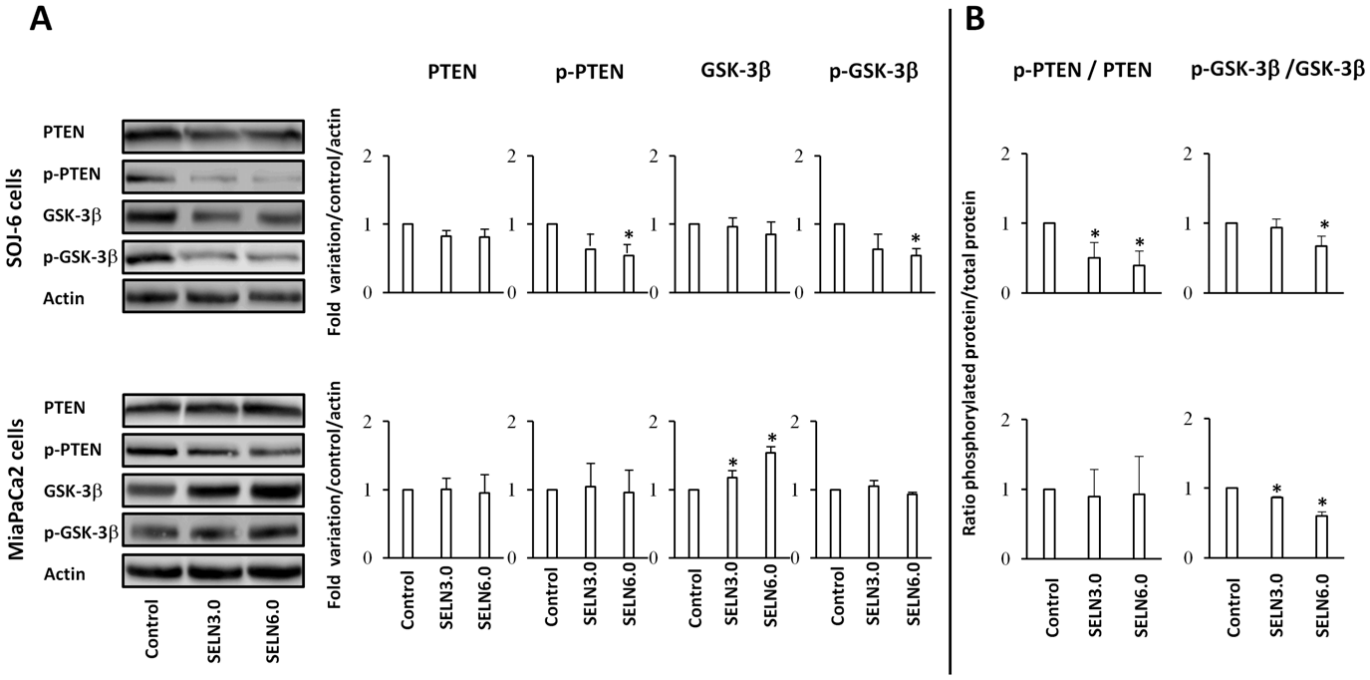
Effect of SELN on PTEN and GSK-3β phosphorylation. (A) SOJ-6 and MiaPaCa-2 cells were starved then treated for 24h with SELN (16 nmoles cholesterol/ml) and lysed. Cell lysate proteins were separated on SDS-PAGE (80 µg of proteins/lane) and electrotransferred onto nitrocellulose membrane. The levels of PTEN, Ser380-phosphorylated PTEN (p-PTEN) GSK-3β, Ser9-phosphorylated GSK-3β (p-GSK-3β) and β-actin were determined by probing membranes with specific antibodies as indicated. Western blots are representative of three independent experiments. Lane 1, control performed in the absence of SELN, Lane 2, in the presence of SELN 3.0, Lane 3, in the presence of SELN 6.0. The right panels in figure 5A represents the quantification of western blots. (B) Ratios of Ser380-phosphorylated PTEN (p-PTEN) to total PTEN and Ser9-phosphorylated GSK-3β (p-GSK-3β) to total GSK-3β determined from A. Results are means (± SD) of three independent experiments using the NIH Image program (Mann-Whitney test).

We have previously shown in SOJ-6 and MiaPaCa-2 cells that PTEN and GSK-3β were phosphorylated consecutively to the constitutive activation of the PI3K/Akt pathway [12]. Furthermore in SOJ-6 cells, blocking the γ-secretase complex resulted in the decrease of Hes-1 expression and of the phosphorylation of PTEN and GSK-3β [12]. Conversely, inhibiting either PTEN or GSK-3β increased Hes-1 expression and partially counteracted the inhibition of proliferation promoted by exosomes, highlighting reciprocal regulations between Notch signaling and PTEN/GSK-3β. Therefore we determined the phosphorylation of both PTEN and GSK-3β upon SOJ-6 and MiaPaCa-2 cells challenging with SELN (**Fig. 5A**). Although PTEN and GSK-3β expressions were not affected by SELN3.0 and SELN6.0, a decrease in PTEN and GSK-3β phosphorylation at residues Ser380 and Ser9 respectively was observed in SOJ-6 cells challenged with SELN6.0 whereas SELN3.0 were ineffective (**Fig. 5A**). Consequently the ratio of Ser380-phosphorylated PTEN to total PTEN and Ser9-phosphorylated GSK-3β to total GSK-3β are significantly decreased in SOJ-6 cells, in particular in SOJ-6 cells challenged with SELN6.0 (**Fig. 5B**). Such modification in PTEN phosphorylation over total PTEN protein upon SELN treatment was not observed in MiaPaCa-2 cells (**Fig. 5A, B**). However the ratio p-GSK-3β over total GSK-3β is significantly decreased in MiaPaCa-2 cells upon challenging with SELN, this apparent decrease in GSK-3β phosphorylation is likely the result of the increased expression of GSK-3β in the presence of SELN (**Fig. 5A** **)**, a result that needs more studies to be explained. Thus SELN6.0, similarly to exosomes from SOJ-6 tumor cells [12] decreased the phosphorylation of PTEN and of GSK-3β leading to functional proteins [**16]**, [17] and drove SOJ-6 cells to the mitochondrial-dependent apoptotic pathway.

### Cell Response to Ceramide-apoptotic Pathway and Endoplasmic Reticulum-stress

**Figure 6:**
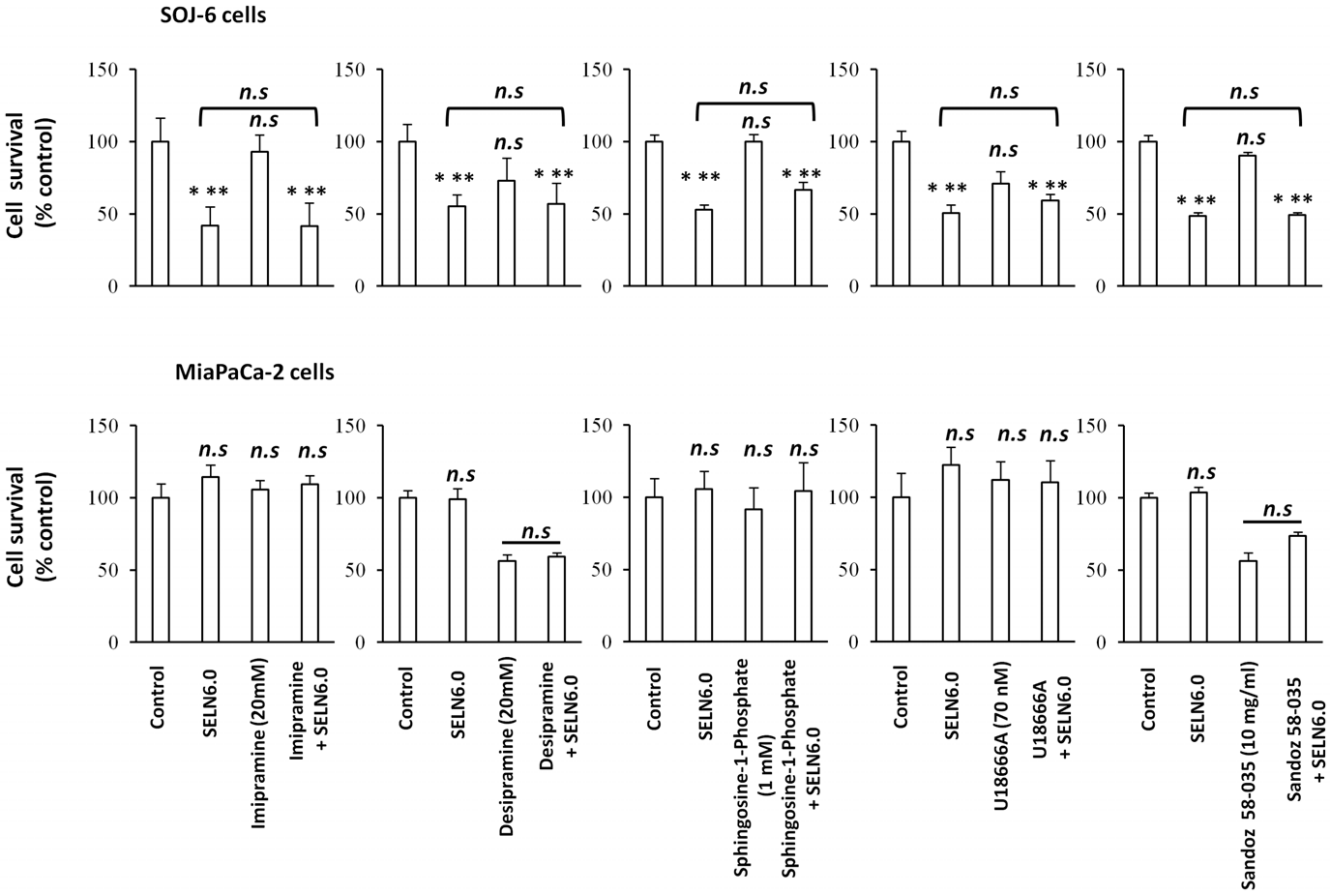
Proliferation of MiaPaCa-2 and SOJ-6 cells in the presence of drugs. MiaPaCa-2 and SOJ-6 cells were incubated 1h with drugs affecting lipid metabolism at the indicated concentration then SELN6.0 (16 nmoles cholesterol/ml) were added for 24h in the presence or absence (control) of drugs at indicated concentration. At the end of incubation, proliferation was measured by MTT assay and expressed as % of control. Results are means (± SD) of independent experiments, (n = 24, Student’s *t*-test).

SELN are lipid-rich nanoparticles including sphingomyelin (SM), cholesterol, and ceramides (Cer) (**Table 1**). SM and ceramides can be internalized by the cell to reach the endo-lysosomal compartment where they can activate the ceramide-dependent apoptotic pathway *via* sphingomyelinase activation [18]. To determine whether the ceramide-dependent apoptotic pathway is involved in apoptosis promoted by SELN6.0 we used inhibitors of this apoptotic pathway. Imipramine and desipramine, two potent inhibitors of acid sphingomyelinase, had no reverse effect on the inhibition of the SOJ-6 cell survival induced by SELN6.0 (**Fig. 6**). Furthermore sphingosine-1-phosphate (S1P), which inhibits the ceramide-induced apoptosis [19] did not affect the decrease in SOJ-6 cell survival observed in the presence of SELN6.0. Collectively these data strongly suggested that the ceramide-dependent apoptotic pathway can be ruled out.

**Figure 7:**
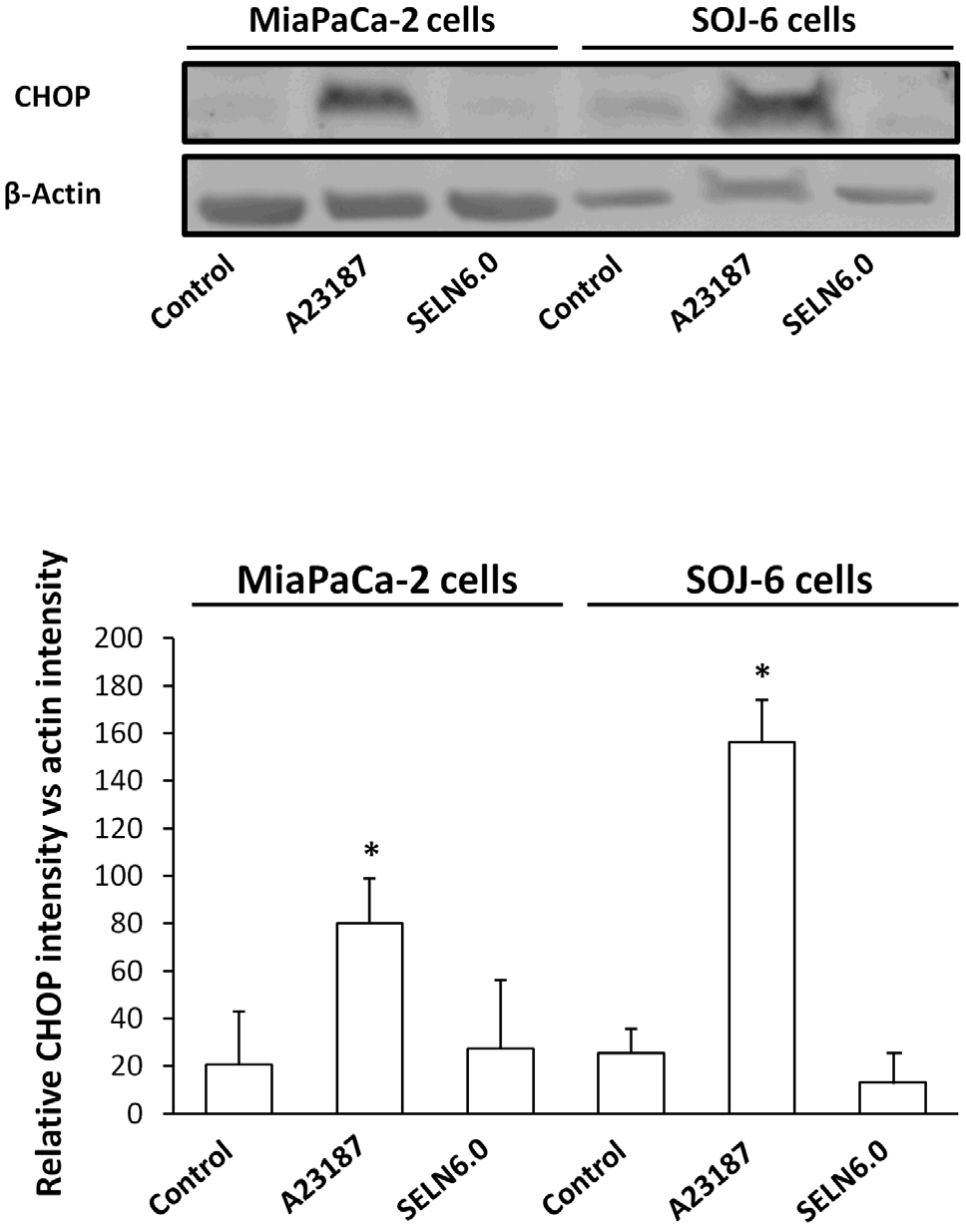
SELN cholesterol and UPR response. MiaPaCa2 and SOJ-6 cells were starved then incubated for 24h in the presence of the UPR inducer calcium ionophore A23187 (2.5 µg/ml), in the presence of SELN6.0 (16 nmoles cholesterol/ml) and in the absence of effector (control). At the end of the incubation cells were lysed and proteins were separated on SDS-PAGE (100 µg of proteins/lane) and electrotransferred onto nitrocellulose membrane. After saturation, membranes were incubated with primary antibodies to CHOP or β-actin and with the POD-labelled antibodies to mouse IgG. The upper part displays a typical western blotting, the lower diagrams are averages of western blotting quantification (± SD) of three independent experiments (Mann-Whitney test).

Normally endoplasmic reticulum (ER) membrane contains low level of free cholesterol [20] and excess of trafficking of free cholesterol to the ER would perturb the ER function. When severe, this stress induces apoptosis [21]. As a consequence free cholesterol loading of macrophages results in the Unfolded Protein Response (UPR) and apoptosis [20]. When used at nanomolar concentration U18666A selectively interferes with cholesterol trafficking to the ER [20]. As shown here U18666A did not significantly affect the effects of SELN6.0 on SOJ-6 cell survival **(** **Fig. 6** **)**. Further the conversion of free cholesterol into cholesteryl ester, an event due to the activity of the Acyl Co-A Acyl Transferase (ACAT) and occurring within the ER, prevents the ER accumulation of free cholesterol [22]. The Sandoz 58035 ACAT inhibitor did not affect the SELN6.0 effects on SOJ-6 cells survival **(** **Fig. 6** **)**. The data obtained with those two drugs indicate that the accumulation of free cholesterol in the ER is not responsible for the effects of SELN6.0 on SOJ-6 cell apoptosis. None of those inhibitors affect MiaPaCa-2 cell survival, excepted Sandoz 58035 and desipramine. However in both cases SELN6.0 did not modify the Sandoz 58035 and desipramine effects. Further we monitored the UPR activity by means of the expression of CHOP (also known as GADD153), an UPR-induced downsteam transcription factor [23]. Contrarily to calcium ionophore A23187 (a well known UPR-inducing drug), SELN6.0 did not affect CHOP expression (**Fig. 7**) thus supporting the notion that cholesterol accumulation in the endoplasmic reticulum (ER) membrane again is not responsible for the effects of SELN on cell fate. These data confirm that neither SM or ceramide nor cholesterol death pathways are involved in SOJ-6 cell death as already found with exosomes [11].

### Interactions of SELN with Cells

**Figure 8:**
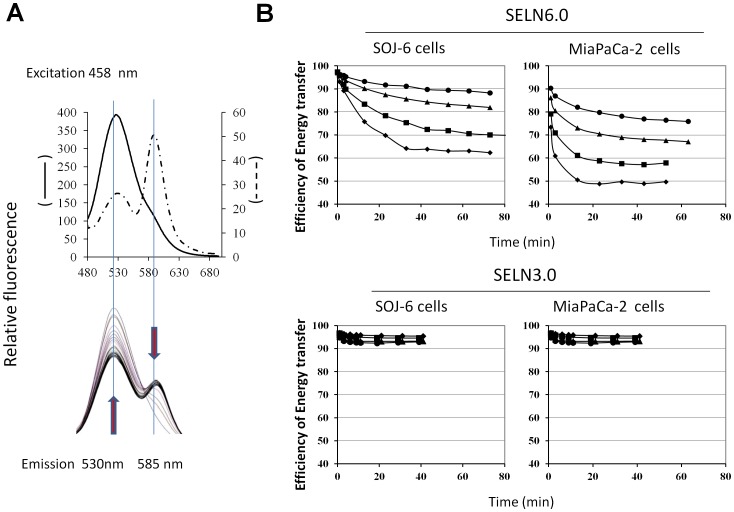
SELN interactions with SOJ-6 and with MiaPaCa-2 cells. (A) Left spectrum depicts a typical FRET obtained with SELN6.0 labeled with *N*-NBD-PE and *N*-Rh-PE. Once excited at 458 nm, *N*-NBD-PE transfers energy to *N*-Rh-PE generating light emission at 585 nm (dashed line curve, upper panel). When SELN6.0 preparation was diluted in a micellar solution of detergent (Tween 20, 1% final) the FRET depicted by the 585 nm emission peak disappears to favor *N*-NBD-PE maximal emission (f_0_) at 530 nm (single line curve, in upper panel). The lower panel shows typical variation of fluorescence emission (following excitation at 435 nm) of SELN6.0 (50 µl) in the presence of 1.0×10^6^ SOJ-6 cells. Arrows indicate variations in fluorescence emission with time (0 min up to 60 min, 3 min steps). (B) Variation with time of the energy transfer efficacy *E* = 1-(f/f_0_) when cells were mixed with SELN. f_0_ is determined at the end of each experiment (see A). Typical data obtained with : • 0.25×10^6^, ▴ 0.5×10^6^, ▪ 1.0×10^6^, and ♦ 2.0×10^6^ cells.

When SELN preparation loaded with 1-oleoyl-2-(6-((7-nitro-2-1,3-benzoxadiazol-4-yl)amino(hexanoyl)-sn-glycero-3-phosphoethanolamine (*N*-NBD-PE) and 1,2-dioleoyl-sn-glycero-3-phosphoethanolamine-N-(lissamine-rhodamine sulfone) (*N*-Rh-PE) was excited at 458 nm, emission peaks culminating at 530 nm (Em530) and at 585 nm (Em585) were observed [24]. The latter peak characteristic of *N*-Rh-PE arises from fluorescence (Föster) resonance energy transfer (FRET) between the couple *N*-NBD-PE donor and *N*-Rh-PE acceptor after *N*-NBD-PE excitation at 458 nm. Fusion/exchange is accompanied by spectral changes that are a decrease in emission peak at 585 nm (Em585) and an increase in emission peak at 530 nm (Em530). Each change is indicative of a reduction in the efficiency of energy transfer between *N*-NBD-PE and *N*-Rh-PE consistent with fusion/exchange of fluorescent vesicles followed by lateral diffusion/exchange of fluorescent lipids in the plane of cell membrane. Upon addition of Tween 20, the emission at 530 nm increased and reached a maximum value after 30 min incubation (likely due to system equilibration, **Fig. 8A**). This value represents f_0_, *i.e*. the emission of *N*-NBD-PE monomer dispersed in detergent micelles and was determined for each SELN preparation. When *N*-NBD-PE and *N*-Rh-PE loaded SELN (6 nmoles cholesterol/ml, a concentration avoiding a decrease in cell survival) were incubated with SOJ-6 and MiaPaCa-2 cells at 25°C, an increase in Em530 and a decrease in Em585 were observed with time showing that the efficiency of energy transfer decreased between donor and acceptor. This decrease was linked to cell amount, being higher with 1.0×10^6^ cells than with 0.25×10^6^ cells (**Fig. 8B**). These data are consistent with a fusion/exchange of SELN, in particular SELN6.0, with SOJ-6 and MiaPaCa-2 cell membranes followed by lateral diffusion and dilution of fluorescent phospholipids within the plane of cell membrane. With regards to SELN3.0 the efficiency of energy transfer reaches a 10% decrease only, independently of cell lines. This last result may suggest that SELN3.0 were not incorporated in cell membranes and/or that no dilution of fluorescent lipids occurred following interaction.

**Figure 9:**
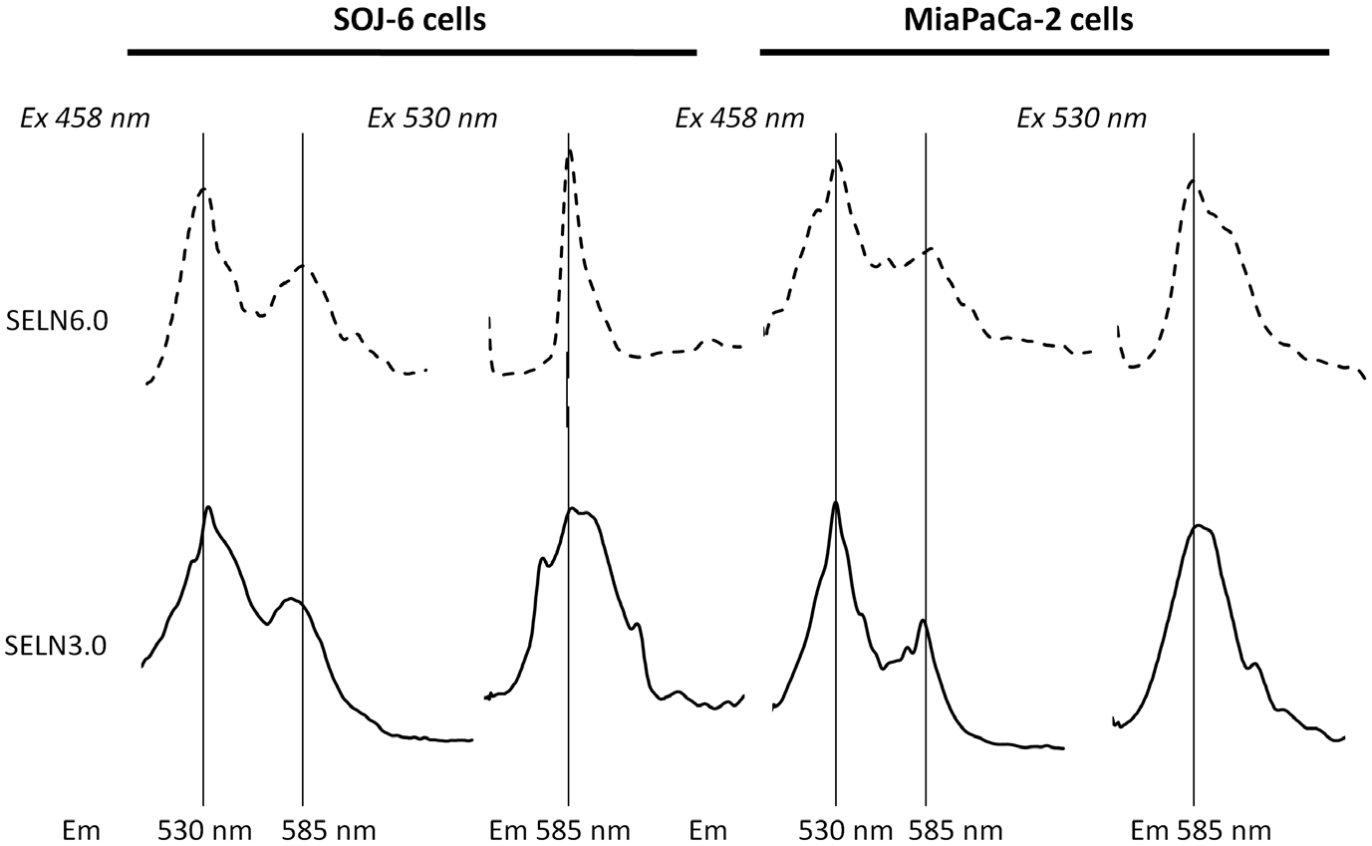
Cell fluorescence upon incubation with fluorescent SELN. SOJ-6 and MiaPaCa-2 cells (0.5×10^6^ cells) were suspended in 2 ml (final volume) of PBS and incubated for 90 min (25°C) with 50 µl SELN3.0 and SELN6.0 (4 nmoles cholesterol/ml). Cells were then pelleted and washed with PBS (twice) and finally suspended in 2 ml PBS before fluorescence analysis after excitation at 458 nm (left peaks) or at 530 nm (right peaks). The self-fluorescence of cells was subtracted from given spectra.

To further demonstrate the fluorescent lipids (*i.e.* SELN) internalization in cells, SOJ-6 and MiaPaCa-2 cells (1.0×10^6^ cells/ml) were incubated with SELN3.0 and SELN6.0 (6 nmoles cholesterol/ml) and extensively washed with PBS. After excitation at 458 nm or 530 nm, emission peaks were detected on either SOJ-6 or MiaPaCa-2 cells after incubation with SELN6.0 or SELN3.0 (**Fig. 9**). These fluorescence emission peaks were centered on 530 nm and 585 nm following excitation at 458 nm and 530 nm respectively. Spectra represent the difference between cells incubated with fluorescent SELN and self-fluorescence of mock-treated cells. These results suggested that SELN6.0 and SELN3.0 were effectively captured by cells. However, residual FRET was observed in cell upon incubation with fluorescent SELN (*i.e.* emission peak at 585 nm after excitation at 458 nm). This would imply that a fraction of fluorescent lipids remained in a close vicinity to allow energy transfer. This would suggest that SELN kept associated with membranes without fusion/exchange or more likely that the incorporation of fluorescent lipids occurred within a restricted area of membranes or within a specific cell compartment.

### Localization of SELN in Cells

**Figure 10:**
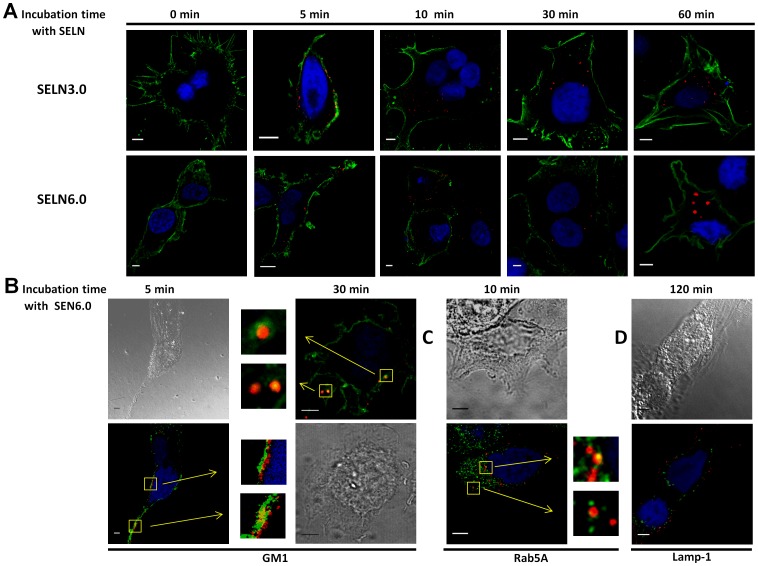
Fluorescent SELN incorporation. (A) SOJ-6 cells were seeded on 1.4 cm-diameter cover slips in 12-wells plate, once adherent cells were starved for 24h before incubation with SELN3.0 or SELN6.0 (8 to 10 µl of SELN solution corresponding to 1.6 nmol cholesterol in 100 µl culture medium). SELN used in this experiment were only loaded with *N*- Rh-PE. Cells were incubated for 0, 5, 10, 30 and 60 min with *N*- Rh-PE-loaded SELN. At the end of the incubation time cells were washed with PBS and then fixed and saturated, nuclei were blue-colored with Draq5 (1 µM, 10 min, 37°C) and saturated with 1% BSA. Cell plasma membranes were further labelled with mAb16D10, a monoclonal antibody which binds to the tumor cell membrane antigen 16D10 [25]. mAb16D10 is then detected using a secondary antibody to IgM, coupled to FITC. Examination was performed using a SP5 Leica confocal microscope (Scale bar = 5 µm). (B) Plasma membrane lipid microdomains were visualized *via* the binding of the cholera toxin subunit B (CT-B) to raft ganglioside GM1. SOJ-6 cells were grown in complete DMEM medium, incubated with SELN6-Rh-PE (5 min, 37°C in FCS-depleted DMEM) before washing twice. Cells were then fixed with PFA, and nuclei were blue-colored with Draq5. Fixed cells were washed and incubated with the CT-B (0.5 µg/ml final concentration, 10 min, 4°C) before washed and incubated with Alexa Fluor 488–conjugated antibodies against CT-B (15 min, 4°C) (GM1, 5 min). To detect the intracellular localization of the CT-B, cells were first incubated with CT-B (see above), washed, and incubated with Alexa Fluor 488–conjugated antibodies against CT-B, before incubation with SELN6.0-Rh-PE, during 30 min at 37°C. Finally cells were fixed with PFA and then nuclei labelled with Draq5 (GM1, 30 min). (C, D) SOJ-6 cells were treated as in A and incubated (for the indicated time) with SELN6.0 labelled with *N*-Rh-PE (1.6 nmol cholesterol/100 µl culture medium). Cells were fixed with PFA and nuclei labelled with Draq5. Cells were permeabilized with saponin (0.1%, 30 min at room temperature), saturated (BSA, 1%, 30 min at room temperature) and incubated with antibodies (C) to early-endosome marker Rab5A, or (D) to late endosome marker Lamp-1 and further detected with an Alexa Fluor 488-labelled secondary antibodies. In B and C, squares indicate co-localization (yellow) and arrows indicate enlarged areas (inserts) where GM1, or Rab5A co-localizes with *N*- Rh-PE labelled SELN6.0 (Scale bar = 5 µm).

**Figure 11:**
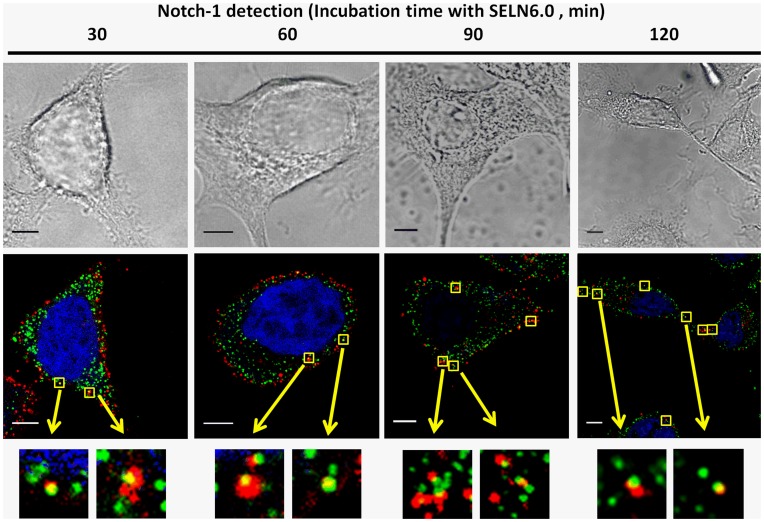
Co-localization of Fluorescent SELN6.0 with Notch-1 in SOJ6 cells. SOJ-6 cells were treated as in **Fig. 10** and incubated (for the indicated times) with *N*- Rh-PE-loaded SELN 6.0 (1.6 nmol cholesterol/100 µl culture medium). Cells were fixed with PFA, permeabilized, saturated and nuclei labelled with Draq5. Cells were then incubated with antibodies to Notch-1 (extracellular domain) and further detected with Alexa Fluor 488-labelled secondary antibodies. Squares indicate co-localization (yellow) and arrows indicate enlarged area (inserts) where Notch-1 co-localizes with *N*- Rh-PE labelled SELN6.0 (Scale bar = 5 µm).

To study the cellular localization of SELN, in part to determine whether SELN localize in cell compartments where the Notch-1 receptor matures, SOJ-6 cells were incubated with SELN3.0 and SELN6.0, loaded with *N*-Rh-PE only for time up to 60 min (a time sufficient to record full events in SELN fusion/exchange; not shown). For a short incubation time (5 to 10 min) *N*-Rh-PE-loaded SELN fluorescence represented by red dots decorated the plasma membrane and co-localized (yellow) with the 16D10 pancreatic-specific membrane antigen [25] (**Fig. 10A**, in green). Thus confocal microscopy images do not support the hypothesis that dilution of SELN fluorochrome occurs within the plasma membrane. However it could be that the fluorescent lipid is dispersed throughout the plasma membrane at undetectable levels and that only highly enriched areas are visible. For a longer incubation time (>30 min), the fluorescent lipid appeared as punctuated figures within the cell cytoplasm. After 60 min incubation, larger spots were observed in particular for SELN6.0, suggestive of accumulation of the fluorescent lipid within intracellular structures. Because Notch-1 receptor localizes in lipid microdomains [26] we next determine whether SELN6.0 also localizes in these lipid structures. For this purpose we labeled lipid microdomains with the cholera toxin subunit B which binds to ganglioside M1 (GM1) in lipid microdomains. As shown in **Fig. 10B** the fluorescent SELN6.0 co-localized with the GM1 at the level of membrane microdomains. After 30 min-incubation time with cells, SELN6.0 was located with GM1 in an area subjacent to the plasma membrane. Interestingly Notch-1 can also be matured in early endosomes [26]. Indeed confocal images suggested that *N*-Rh-PE-loaded SELN6.0 co-localizes with the early endosomes marker Rab5A (**Fig. 10C**). However SELN6.0 did not reach late endosomes as *N*-Rh-PE-loaded SELN never co-localized with Lamp-1 late endosomal marker (**Fig. 10D**) even after long time incubation. Finally we showed that following 30 min incubation, *N*-Rh-PE-loaded SELN co-localized with Notch-1 in membrane structures. For longer incubation time SELN6.0 and Notch-1 co-localized in sub-membrane structures (**Fig. 11**). As above suggested membrane structures could be lipid microdomains and sub-membrane localization may be representative of a co-localization of SELN6.0 with early endosomes.

## Discussion

In a recent publication we have hypothesized that exosome lipids are key-factors in the induction of target cell death [12]. In the present study we investigated the effects and roles of these lipids on cell fate. Exosomes are constitutively enriched in proteins (http://exocarta.ludwig.edu.au), which may participate in multiple cell signaling, but the role of lipids constitutive of exosomes is poorly investigated. However the crucial role of lipids was suggested by our previous studies [11], [12] because after trypsin and endoglycoceramidase treatments or heating, exosomes from SOJ-6 cells were still able to decrease cancer cell proliferation. This allows us to rule out the implication of protein ligands associated with exosome membrane such as heat shock proteins and CD91 [11], among others. It is also worthy of note that Fas-ligand was not detected on exosomes expressed by tumor pancreatic SOJ-6 cells (unpublished data). In SOJ-6 cells, lipids constitutive of liquid ordered phase (Lo) were detected in lower amount than lipids forming liquid disordered phase (Ld) [11]. However, lipids constitutive of ordered membrane microdomains, SM and cholesterol, are in much larger amounts in exosomes than in SOJ-6 parent cells [11], which appears to be a general feature of exosomes [10], [27], [28].

Among the different lipid classes detected in exosomes, some could be of importance in the inhibition of cell proliferation in response to exosome challenging. It is known that lysophosphatidylcholine can be processed by G2A receptor to impact on dendritic cell fate [29]. Whereas lysophosphatidylcholine was found in exosomes from SOJ-6 cells, it could not be detected in those from BxPc-3 cells (unpublished data), yet both cell exosomes inhibited cell proliferation [11], [12]. Phosphatidylserine (PS), a minor components of exosomes likely exposed at their surface [30], may participate in engulfment of the exosomes by means of PS receptors [30] such as Tim-4 [31], to induce cholesterol accumulation and death of monocytes [32]. However, it is not known whether PS receptors/ligands are expressed by pancreatic cancer cells. Nonetheless, to demonstrate the implication of exosomal lipids in promoting the death of SOJ-6 pancreatic tumor cells, we synthesized exosome-like nanoparticles or SELN solely composed of lipids among which lipid-forming microdomains, SM, ceramide and cholesterol predominate. Ceramide and SM can be internalized by the cell to reach the endo-lysosomal compartment where they can activate the ceramide-dependent cell death pathway by means of sphingomyelinase activation [18]. Also cholesterol can promote the endoplasmic reticulum stress and the UPR with consecutive cell death [20]. However, we demonstrated that both death pathways depending on SM/ceramide metabolism and on cholesterol accumulation inducing endoplasmic reticulum stress and UPR can be ruled out. We have shown that SELN induced a decrease in SOJ-6 cell survival and promoted cell death thus mimicking the effects of exosomes [11], [12]. This means that exosome lipids alone could be responsible for the death of tumor pancreatic SOJ-6 cells. To further establish the involvement of lipid microdomains (*i.e.* lipids forming liquid ordered phase) in these biological effects, we increased the ratio Lo/Ld (*i.e.* raft lipids versus non-raft lipids) from 3.0 up to 6.0. These SELN were able to decrease the SOJ-6 cell proliferation in a concentration-dependent manner. Because higher the ratio Lo/Ld (*i.e.* the amount of raft lipids), greater the detrimental effects on SOJ-6 cells, we can conclude that the raft lipids of SELN are linked to the ensuing cell death.

Recent studies have revealed that exosomes may represent means for the transfer of biological material from cell to cell by binding to or fusion/exchange with plasma membrane, resulting in the delivery of signals affecting the cell phenotype [33]. This delivery may also result in lipid incorporation in membrane of target cells and in consecutive membrane dysfunction. Membrane microdomains display sizes ranging from 10 to 200 nm, and are heterogeneous in composition. They are highly dynamic sterol- and sphingolipid-enriched domains that compartmentalize important cellular processes and represent platforms for cell signaling where multiple signals are initiated [34]. The size of SELN, which appears stable with time, could be compatible with a fusion/exchange with lipid microdomains in cell membrane, a notion that is supported by our FRET experiments. Our confocal microscopy data indicate that SELN6.0 interacted with cell membranes at the level of lipid microdomains and were internalized with GM1, a marker of lipid microdomains. Co-localization of fluorescent SELN with the CT-B/GM1 and with Rab5A (a marker of early endosomes) fits with two hypothetic mechanisms. The first one is the fusion/exchange of lipid SELN with microdomains of the plasma membrane followed by endocytosis. The second one is endocytosis followed by the fusion/exchange of SELN with microdomains of the endosomal membrane. Because SELN co-localize with Notch-1 and with the 16D10 antigen at the level of plasma membrane and with Notch-1 and Rab5A at the level of early endosomes, we believe that these two hypotheses are not exclusive. The residual FRET observed in cells once challenged with *N*-NBD-PE and *N*-Rh-PE loaded SELN suggests that the fusion/exchange occurs in restricted area of the membrane that might be lipid microdomains present in plasma and endosome membranes. However fluorescent phospholipids were never found co-localized with the Lamp-1 positive late endosomal compartment, meaning that lipids never reach the lysosomal compartment [35]. Further a study has concluded that exosomes are endocytosed and accumulate in endocytic compartments [36]. Therefore SELN appeared to impact on cell behavior following interaction with lipid microdomains.

Further we provided evidence that Notch-1 in SOJ-6 cells is a likely cell target of exosomes [12]. Notch-1, ADAM17 (a α-secretase), and the γ-secretase complex are localized in membrane rafts [37]–[39]. The γ-secretase releases Intracellular Notch-1 (ICN), which translocates to the nucleus to induce Hes-1 expression and cell survival [37]. The activities of the γ-secretase and of the α-secretase are shown to be directly and potently affected by their lipid microenvironment in particular by the levels of cholesterol [13], [38], [40]. Exosomes themselves rich in lipid forming rafts may fuse with the membrane of target cells to affect lipid microdomains functioning [41] and the Notch-1 pathway [11]. It is known that Notch-1 can indistinctly be matured at the plasma membrane or in endosomes to release ICN [42]. Endocytosis of Notch receptor is crutial for Notch signaling ability [43]. We showed here that SELN6.0, which co-localized with Notch-1 in plasma membrane and endosomes of SOJ-6 cells, decreased the cell level of ICN and increased the level of Notch-1, suggesting that the γ-secretase activity is affected by SELN, a result that correlates with the regulation of the γ-secretase by its lipid environment in lipid microdomains [13]. Consequently the expression of Hes-1, the nuclear target of ICN, also decreased. Challenging SOJ-6 cells with SELN6.0 activated the caspase 9 and increased the expression of the pro-apoptotic Bax, detrimental to that of anti-apoptotic Bcl-2 proteins. We have shown that challenging SOJ-6 cells with exosomes decreased the Ser380 phosphorylation of PTEN [12] leading to its activation [17]. Furthermore once activated PTEN can directly decrease the Ser9 phosphorylation and promote the activation of GSK-3β to repress Hes-1 expression [12]. Such decrease in phosphorylation and activation of PTEN and GSK-3β also occurred in SOJ-6 cells challenged with SELN of high Lo/Ld ratio. Therefore SELN, in particular SELN6.0, as amply demonstrated with exosomes [12], likely affects the γ-secretase complex functioning to inhibit the Notch-1 survival pathway leading to the activation of PTEN and GSK-3β, the decrease in Hes-1 expression and drives SOJ-6 cells towards the mitochondrial-dependent apoptotic pathway. Regarding the fate of tumor MiaPaCa-2 cells that are poorly sensitive to exosomes, they remained insensitive to SELN as well. It has been hypothesized that the origin of this insensitivity could reside, at least partly, in the high level expression of Notch-1 partners by MiaPaCa-2 cells [12]. Modulating Notch-1 efficiency or expression by means of γ-secretase inhibitor or siRNA specific for Notch-1 sensitize MiaPaCa-2 cells to SELN which therefore agree with this hypothesis. Nonetheless, the difference in sensitivity to SELN could be due to the heterogeneous genetic backgrounds of SOJ-6 and MiaPaCa-2 cells. For instance PTEN mutations have been involved in the resistance to Notch inhibition [43]. However PTEN (a partner of Notch, [11]) is rarely mutated in pancreatic adenocarcinoma and pancreatic tumor cells including SOJ-6 and MiaPaCa-2 cells expressed wild-type PTEN [12]. These two cell lines also expressed wild-type GSK-3β [12]. Notch also represses stem/progenitor cell expansion *via* p53 tumor suppressor protein [44]. Consequently expression of mutated or non-mutated p53 by tumor pancreatic SOJ-6 and MiaPaCa-2 cells could be the source of observed differences in SELN effects. Nevertheless p53 mutations have been observed in DNA specific binding domain in both exosome-sensitive SOJ-6 [45] and exosome-insensitive MiaPaCa-2 tumor cells (V. Sbarra and E. Mas, personal communication). Finally, SELN effects on cell death depend on one side on the use of the Notch-1 signaling pathway as a survival pathway by the target cell and on the other side on lipid composition of SELN and by extension on lipid composition of exosomes, which seems to depend upon cell origin [27], [28]. SELN and exosomes lipids may affect the lipid composition of microdomains where the Notch partners locate in part the γ-secretase complex, thus affecting Notch-1 functionality.

## Materials and Methods

### Materials

Antibodies to Bax, peroxidase (POD)-labelled antibodies to rabbit immunoglobulins (IgG), antibodies to PTEN, to (Ser380)phospho-PTEN, to GSK-3β, to (Ser9)phospho GSK-3β, and antibodies to CHOP were from Cell Signaling (Beverly, MA). Antibodies to Hes-1 and ICN (activated intracytoplasmic Notch form) were from Abcam (Cambridge, UK). Antibodies to Rab5A and to Lamp-1 were kind gifts from Dr E. Ghigo (Urmite, Marseille, France). Antibody to Bcl-2 came from Dako (Glostrup, Denmark). Imipramine, desipramine, Sandoz-58035, calcium ionophore A23187, POD-labelled antibodies to mouse IgG, FITC-labelled antibodies to mouse IgM and antibodies to actin were from Sigma (St Louis, MO). Alexa-Fluor-labelled antibodies to mouse and to rabbit IgG were from Invitrogen (Illkirch, France). U18666A was from Calbiochem (La Jolla, CA). RPMI 1640, DMEM cell culture media, penicillin, streptomycin and trypsin-EDTA were from InVitrogen (Carlsbad, NM). Antibodies to Notch-1 extracellular domain used in confocal studies were from Neomarker (Fremont, CA). Caspase inhibitors were from Alexis (San Diego, CA). Antibodies to Notch-1 (extracellular domain), Notch-1 siRNA mix (sc-36095) and control (scramble) siRNA were from Santa Cruz Biotechnology (Santa Cruz, CA). Sphingosin-1-phosphate, sphingolipids and phospholipids were purchased from Avanti Polar Lipids Inc (Alabaster, AL) and all other lipids (pure grade) were from Sigma-Aldrich (St Quentin-Fallavier, France) unless stated otherwise. Cells lines (BxPc-3, MiaPaCa-2 and HEK 293T) used in this study came from the American Type Culture Collection (ATCC, Rockville, MD). SOJ-6 cells were a gift from Dr M-J. Escribano (INSERM UMR 911, Marseille, France) [46].

### Cell Growth and Cell Survival

Cell lines originating from human pancreatic (adeno)carcinoma were grown in either RPMI 1640 (SOJ-6 cells) or in DMEM (MiaPaCa-2 cells) medium with 10% fetal calf serum (FCS) at 8,000 cells/well unless otherwise stated were seeded in a 96-well culture plates. Cells were then deprived in FCS for 24 h and these quiescent cells were further treated with increasing amounts of effectors, in the absence of FCS. Cell survival was assessed by 3-(4,5-dimethythiazol-2-yl)-2,5-diphenyl (MTT) assay. All determinations were compared to those of cell controls without added effectors and taken as 100%. Results are given as mean ± SD.

### Gamma-secretase Inhibitor IC_50_ Determination

In order to obtain the concentration of γ-secretase inhibitor (GSI) necessary for 50% of inhibition of cell survival (IC_50_), MiaPaCa-2 cells were grown in 96 wells plates (8 000 cells per well). Then cells were starved during 24 hours prior incubation with increased concentrations of GSI (0 up to 50 µM). Finally, 24 hours later MiaPaCa-2 cells were submitted to a MTT test.

### Synthetic Exosome-like Nanoparticles (SELN)

Sphingomyelin (SM) solution constituted of 9 part of chicken egg SM and 1 part of bovine brain SM were saved in stock solution (1 mg/ml) in chloroform. Ceramide (Cer) was a mix of Cer 16∶0, Cer 24∶0 and Cer 24∶1 in chloroform (1∶1∶1 by weight, 1 mg/ml). All other lipids (1 mg/ml) were in chloroform solution. Lipid solutions were mixed to reach concentrations given in **Table 1**. Mix were done to get ratio of total lipids forming ordered phase (Lo) over total lipids forming disordered phase, (Ld) from 3 to 6. [^3^H]-cholesterol (Amersham, SA = 10 700 cpm/µg) was used as tracers to label SELN and to calibrate the used amounts of SELN with exosome quantities [11], [12]. After mixing, the solvent (less than 300 µl) was dried overnight under air-stream and negative pressure. The lipid pellet was further dry under vacuum for at least 1 h to totally eliminate eventual trace of solvent. Lipids were then suspended in PBS (10 mM) pH 7.4 buffer, and sonicated (3 min, 20 W). After slow cooling to room temperature, SELN suspensions were filtered through out a 0.10 µm, 13 mm diameter filter (Durapore VVLP, Millipore, Molsheim, France) and immediately used.

### Fluorescent Labelling of SELN

Fluorescent SELN were synthesized as above after incorporating 0.1–0.2% (total lipid weight) of fluorescent PE. Two probes were incorporated in SELN;1-oleoyl-2-(6-((7-nitro-2-1,3-benzoxadiazol-4-yl)amino(hexanoyl)-sn-glycero-3-phospho-ethanolamine (*N*-NBD-PE) and 1,2-dioleoyl-sn-glycero-3-phosphoethanolamine-N-(lissamine-rhodamine sulfone) (*N*-Rh-PE). Both fluorescent PE are from Avanti Polar Lipids (Alabaster, Al). The ratio *N*-NBD-PE/*N*-Rh-PE of 5/1 to 5/2 allows fluorescence (or Föster) resonance energy transfer (FRET) and the monitoring of fusion/exchange of SELN with cell membrane. When SELN preparation containing both fluorescent probes is excited at 458 nm, emission at 530 and 585 nm are observed [22]. The latter peak characteristic of *N*-Rh-PE arises from fluorescence energy transfer after *N*-NBD-PE excitation at 458 nm. Fusion/exchange is accompanied by spectral changes that are a decrease in emission peak at 585 nm (Em585) and an increase in emission peak at 530 nm (Em530). Each change is indicative of a reduction in the efficiency of energy transfer between *N*-NBD-PE and *N*-Rh-PE consistent with fusion/exchange of fluorescent vesicles followed by lateral diffusion/exchange of fluorescent lipids in the plane of cell membrane. This process lowers the surface density of the energy acceptor *N*-Rh-PE and consequently decreases the efficiency of the energy transfer compared to starting fluorescent SELN. Under the used conditions when SELN are excited at 458 nm, essentially all the fluorescence at 530 nm comes from *N*-NBD-PE. Thus the % of efficiency energy transfer (*E(%*)) which decreases upon fusion/exchange is defined by the relationship [22] *E(*%) = (1– (f/f_0_)) x100, where f is the fluorescence at 530 nm (*N*-NBD-PE) and f_0_ is the fluorescence at 530 nm in the presence of 1% Tween 20. This detergent destroys vesicle structure with the dilution of fluorochromes in a micellar system. Consequently energy transfer between fluorescent molecules is annihilated. Fluorescence spectra were recorded on a LS45 spectrofluorometer (Perkin Elmer, Courtaboeuf, France).

### Cell Transfection

Stable transfection of HEK (Human Embryonic Kidney) 293T cells at 60–80% confluence in DMEM (10% FCS) was performed with pEGFP-C1 and pEGFP-ICN plasmids expressing either fluorescent protein EGFP or EGFP and the active intracellular domain of Notch-1 (both plasmids are gifts from Dr. Freddy Radtke, Epalinges, Switzerland) using the JetPrime-mediated PolyPlus transfection kit according to the manufacturer’s instructions (Illkirch, France). HEK 293T cells were incubated with transfection medium at 37°C in 5% CO_2_ for 6–12 h, then the medium was removed and cells were incubated in 10% FCS-DMEM. Cells were used after 24 h when transfection reaches its maximum as assessed by fluorescent EGFP. Cells were then seeded in 96-well culture plates as above and cultured for 24 h in 0.1% FCS DMEM medium. Quiescent HEK 293T cells were challenged with SELN and cell survival was finally assessed by MTT.

### Notch-1 siRNA Transfection

MiaPaCa-2 cells were plated in 6-well tissue culture plates at a density of 3×10^5^ cells/well. Prior transfection, the culture medium was removed and replaced with OPTI-MEM culture medium (Gibco, Carlsbad, CA). Cells were transfected either with the mix of Notch-1 siRNA or the control siRNA at a concentration of 100 nM, using Oligofectamin (Invitrogen) according to manufacturer’s instructions. After 6h of incubation, FCS was added to the media (10% final) and 24 hours after transfection 16 000 cells were seeded in 96-well culture plates, as soon as cells became adherent they were starved one night. Then, 48 hours after transfection, cells were challenged with SELN6.0 during 24 hours. Finally cell survival has been assessed through a MTT test (Notch-1 knock-down is observed at least during 72 hours after transfection).

### Confocal Microscopy

Cells were seeded in appropriate medium on cover-slips in 12 well-plates (BD Falcon, Le Pont-de-Claix, France). Once adherent cells were starved for 24h and incubated at 37°C with *N*-Rh-PE-loaded SELN. Cells were fixed (paraformaldehyde, PFA, 2% in PBS, 37°C, 15 min) and saturated (bovine serum albumin, BSA, 1% in PBS, 30 min). The cells were then incubated successively with the mAb16D10 primary antibodies for 90 min to label cell plasma membrane [23] and then with secondary antibody to IgM coupled to FITC for 45 min. All the later stages were carried out at 4°C. Plasma membrane lipid microdomains were visualized *via* the binding of the cholera toxin subunit B (CT-B, Vybrant® lipid raft labeling kit, Molecular Probes, Eugene, OR) to raft ganglioside GM1. For this purpose SOJ-6 cells were grown in complete RPMI 1640 medium, incubated with SELN6.0-*N*-Rh-PE during 5 min (in RPMI 1640 depleted in FCS, 37°C) before washing twice with PBS. Then cells were fixed with PFA, washed and incubated according to the manufacturer with the CT-B (10 min, 4°C) before washed and incubated with Alexa-Fluor 488–conjugated antibodies against CT-B (15 min, 4°C). To detect the intracellular localization of the CT-B, cells were first incubated with the CT-B (see above), washed, and incubated with Alexa-Fluor 488–conjugated antibodies against CT-B, before incubation with SELN6.0-*N*-Rh-PE, during 5 or 30 min at 37°C. Finally they were fixed with PFA. For Rab5A, Lamp-1 and Notch-1 localization, cells were incubated with *N*-Rh-PE SELN6.0 for times as indicated then fixed with PFA (see above) permeabilized (0.1% saponin in PBS, 30 min, room temperature), saturated (BSA 1%, 30 min) and incubated with antibodies to the extracellular domain of Notch-1, to Rab5A and to Lamp-1 and detected with Alexa-Fluor 488-labelled secondary antibodies. In each experiment, the cell nuclei were labelled 10 min at 37°C with 1 µM Draq5, a far-red fluorescent DNA dye (Biostatut Limited, Shepshed, UK). For observations, Confocal Laser Scanning Microscopy experiments were performed using a Leica SP5 microscope coupled with a Leica scanning device (Leica Microsystems, Mannheim, Germany). The inverted microscope was equipped with a Plan-Apochromat 63× objective (NA = 1.4). Images were recorded with LAS AF Lite acquisition software and were calculated with the public-domain ImageJ software (NIH; http://rsb.info.nih.gov/nih-image/). Each image was represented with 1024×1024 pixels measuring 70×70 nm^2^ each on average, and recorded with a frame mode to reduce background noise (average on three scanning images). Image acquisition was performed with the Confocal Laser Scanning Microscope (CLSM) spectral mode selecting specific domains of the emission spectrum, *i.e.* FITC was excited at 488 nm with an argon laser and its fluorescence emission was collected between 500 and 530 nm.

### Monitoring Caspase Activation

SOJ-6 cells grown in 8-well plates (BD Falcon) were treated with SELN for 24 h prior to the addition of CaspACE FITC-VAD-fmk *in situ* marker (Promega, Charbonnière, France) at a final concentration of 10 µM in the culture medium according to manufacturer’s instructions. Then cells were washed in PBS, fixed (15 min, 37°C) in 2% paraformaldehyde, and washed once again. The number of fluorescent cells was determined in triplicate on collection of 10 fields randomly examined under a fluorescence microscope (Axiover, Carl Zeiss, Iena, Germany). When stated, cells were pre-incubated for 4 h with caspase inhibitors (4 µM) before treatment with SELN.

### Apoptosis Determination

Apoptosis was determined using the Terminal Transferase dUTP Nick End Labeling (TUNEL) assay to detect DNA degradation in apoptotic cells. For this purpose ApopTag**®** Red In Situ Apoptosis Detection Kit (Merck, Millipore) was used. Briefly, fragmented DNA was stained according to the protocol given with the kit. Nuclei were counterstained with diamidino-2-phenylindole (DAPI). Apoptotic cells were visualized under a Zeiss fluorescence microscope equipped with a digital camera. The ratio of apoptotic cells to total cells was counted in 5-to-10 random fields representing 600-to-800 cells of three independent experiments.

### Sucrose Density Gradient

SELN loaded with trace amount of [^3^H]-cholesterol were suspended in 0.5 ml of Hepes buffer (20 mM, pH = 7.4) supplemented with sucrose to reach a density of 2.5 g/ml. Twelve ml of a linear gradient of sucrose (0.25–1 M) density was layered on 1 ml of SELN suspension and ultracentrifuged [11]. Fractions (1 ml) were collected and radioactivity counted. The density of each fraction was determined by refractometry.

### Electron Microscopy

Three µl of freshly prepared SELN was examined by electron microscopy to determine their size. SELN were disposed on top of Formvar-coated 300-mesh carbon grids and treated as described [11]. Nanoparticles on grids were examined and pictures were captured at 0, 24 and 48 h, using the built-in microscope (JEM 1400, Jeol, Croissy/Seine, France) software and treated with Image J software to measure SELN diameters.

### SDS-PAGE and Western Blottings

After treatment, the cells were washed three times with ice-cold PBS, harvested and pelleted by centrifugation. Pellets were washed twice and lysed at 4°C in 0.5 ml of lysis buffer (10 mM Hepes pH 7.4, 200 mM NaCl, 1.5% Triton X-100, 5 mM EDTA, 2.5 mM MgCl_2_, and 2 mM CaCl_2_, protease inhibitors, (Complete TM, Roche Diagnostics, Meylan, France) and phosphatase inhibitors cocktail (Sigma). After lysis, homogenates were sonicated (10 sec, 40 W, 4°C) clarified by centrifugation at 10, 000×g for 15 min at 4°C. An aliquot was saved for protein determination using the bicinconinic acid (µBCA) assay (Pierce, Rockford, IL). Proteins in reducing SDS buffer were separated on 10% polyacrylamide gels and 0.1% SDS. After electrophoretic migration, proteins were transferred onto nitrocellulose membranes and processed for immunoblotting by using appropriate primary and POD-labelled secondary antibodies. After washes, membranes were developed with a chemoluminescent substrate [11].

### Lipid Analysis

Lipids were extracted in chloroform-methanol-water (1∶2∶0.9, v/v/v) in a Dounce homogenizer in the presence of standards and then analyzed by gas liquid chromatography for phosphatidylcholine and cholesterol mass content as already described [11], [47].

### Statistical Analysis

Each experiment was done at least three times and results expressed as means ± SD. Difference between experimental groups were analyzed with the Student’s *t*-test or the Mann-Whitney test as specified in the legend of figures. Significance was set as (*) *P*≤0.05; (**) *P*<0.01 and (***) *P*<0.001. *n.s.* stands for not significant difference.

## Supporting Information

Figure S1 **Gamma secretase inhibitor IC_50_ determination.** MiaPaCa2 cells were grown in complete medium in 96 wells plates (8000 cells per well). Then cells were starved 24 hours before incubation with different concentrations of L-685,458 γ-secretase inhibitor (GSI) from 0 to 50 µM. After 24 hours cells were submitted to a MTT test to determine cell survival. IC50 was calculated as average ± SD of three independent experiments. Each value of each experiment is the average of 10 point measurements.
(TIF)
